# Management of small bowel volvulus in a patient with simultaneous pancreas-kidney transplantation (SPKT): a case report

**DOI:** 10.1186/1752-1947-1-106

**Published:** 2007-09-28

**Authors:** Unal Aydin, Pinar Yazici, Huseyin Toz, Cuneyt Hoscoskun, Ahmet Coker

**Affiliations:** 1 Ege University School of Medicine, Organ Transplantation and Research Center, Izmir, Turkey

## Abstract

There are several surgical complications which can occur following simultaneous pancreas-kidney transplantation (SPKT). Although intestinal obstruction is known to be a common complication after any type of abdominal surgery, the occurrence of small bowel volvulus, which is one of the rare causes of intestinal obstruction, following SPKT has not been published before. A 24-year-old woman suffering from type I diabetes mellitus with complications of nephropathy resulting in end stage renal disease (ESRD), neuropathy and retinopathy underwent SPKT. On the postoperative month 5, she was brought to the emergency service due to abdominal distention with mild abdominal pain. After laboratory research and diagnostic radiological tests had been carried out, she underwent exploratory laparotomy to determine the pathology for acute abdominal symptoms. Intra-operative observation revealed the presence of an almost totally ischemic small bowel which had occurred due to clockwise rotation of the mesentery. Initially, simple derotation was performed to avoid intestinal resection because of her risky condition, particularly for short bowel syndrome, and subsequent intestinal response was favorable. Thus, surgical treatment was successfully employed to solve the problem without any resection procedure. The patient's postoperative follow-up was uneventful and she was discharged from hospital on postoperative day 7. According to our clinical viewpoint, this study emphasizes that if there is even just a suspicion of acute abdominal problem in a patient with SPKT, surgical intervention should be promptly performed to avoid any irreversible result and to achieve a positive outcome.

## Background

Small bowel volvulus (SBV) is rarely encountered in adults. SBV generally occurs in a normal abdominal cavity and its etiology is still not clear. There are two categories of small bowel volvulus: a) primary small-bowel volvulus, in which there are no predisposing anatomical abnormalities, and b) secondary small-bowel volvulus, in which an acquired or a congenital abnormality causes rotation of the bowel [1]. There are a few reports in the literature that small bowel volvulus occurs as a late postoperative complication. The reported operations include gastrostomy [2], gastrectomy [3] and total hip replacement [4]. In this case report, a young woman underwent exploratory laparotomy due to small bowel volvulus after simultaneous pancreas-kidney transplantation (SPKT). This is the first report in the literature of small bowel volvulus secondary to SPKT.

## Case presentation

**Figure 1:**
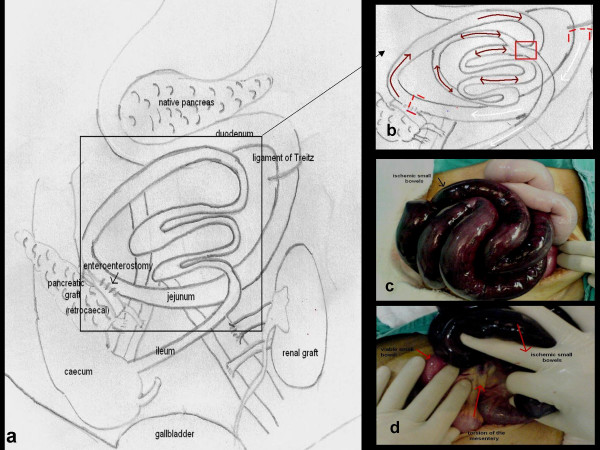
**a: **The diaphragmatic representation of the SPKT performed in this case. Enteric drainage for pancreatic graft was carried out. **1b: ** Diaphragmatic representation of the anatomy found at re-operation. Initiation of the ischemic intestinal segment was from the beginning of the enteroenterostomy (brown arrows with double endings), white arrows indicated the healthy intestine between the red square brackets 30 cm distal to the Treitz ligament. The point of the torsion of the mesentery was shown in red square. **1c: **Ischemic small intestinal segment secondary to volvulus. **1d: **Torsion of mesentery of the small intestine

**Figure 2:**
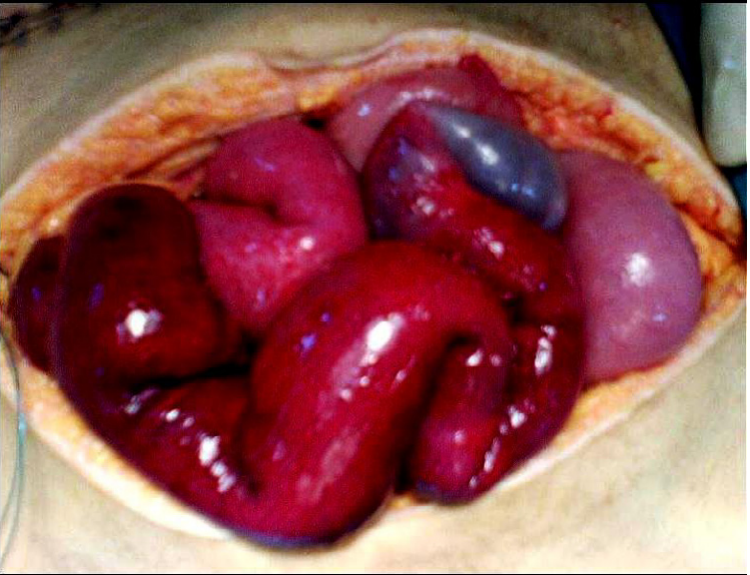
View of the small intestine with a good blood supply, after derotation procedure.

A 24-year-old female with a 14-year history of type I diabetes mellitus that was complicated by nephropathy, neuropathy, and retinopathy underwent SPKT at Ege University School of Medicine's Organ Transplantation Center. In the fifth postoperative month, she was admitted to the hospital with progressive abdominal distension and mild pain. The patient's prior surgical history indicated that her pancreas was located in the retroperitoneal region in the right iliac fossa and her kidney was located to the left. After Y graft interpositioning was performed between the splenic and superior mesenteric artery of the pancreatic graft on the back-table, anastomosis was performed on the right external iliac artery. The portal vein of the graft was end-to-side anastomosed to the recipient external iliac vein. The duodenal part of the graft, which was transected on both sides with circular stapler, was side-to-side anastomosed to the recipient jejunum 30 cm distal to the Treitz ligament (Figure 1a). After completing the transplant of the pancreas, the kidney transplant was performed on the left side. Although both grafts functioned adequately in the early postoperative period, the patient had an acute abdominal episode occurring with mild abdominal pain, distension, nausea and vomiting on the fifth postoperative month. The physical symptoms included tachycardia (120–130 beats/min) and low blood pressure of 90/45 mmHg. Other findings included tenderness, decreased bowel sounds and abdominal distension, which was particularly located in the periumbilical region with palpable, dilated intestines. Laboratory findings indicated leucocytosis (17.9 × 10^3^/mm^3^), high serum lactate dehydrogenase (852 U/L) and high D-dimer (532 mcg/L) levels. A plain upright radiography of the abdomen was found to be non-specific. Abdominal ultrasonography indicating dilatation and edema of the small intestine, and abdominal tomography were then performed. Both revealed minor intestinal dilatation without determining the primary cause. Seventeen hours after admission to the hospital, we performed exploratory laparotomy. During the operation, we observed that almost the entire small intestine was gangrenous, but only 40 cm at the proximal part had adequate blood supply. The distal segment of the small intestine seemed to be ischemic due to clockwise rotation of the mesentery (Figure 1b–c–d). We therefore performed counter-clockwise rotation of the torsioned intestinal segment in order to restore the original anatomic position. After identifying and solving the primary problem, the temperature of both the abdominal region and the ischemic small intestine were kept at a level of 37°C by wrapping them with warm swabs. The whole serosal surface of the intestine was also packed with warm, wet gauze pads. In addition, papaverine was directly applied to the ischemic intestinal segment. After waiting for almost thirty seconds in this position, reexamination revealed excellent blood supply to the small intestine (Figure 2). Because resectional surgery posed a risk of the development of short bowel syndrome for this case, the operation was completed without any additional intervention. After it was determined that laboratory results continued at normal levels, that there was enough oxygenation in the arterial blood gases and that bowel function was normal, the patient was discharged on the postoperative day 7 without any complications. Her follow-up period was arranged according to a checkup schedule for SPKT procedures. The patient has been well for almost one year and no recurrence has been observed.

## Discussion

Small bowel volvulus is a rare but life-threatening medical emergency. Bowel infarction can only be prevented by applying prompt preoperative investigation and immediate surgical intervention, which is the only way to overcome the problem successfully. The most frequent related conditions for secondary small bowel volvulus are bands, adhesions, Meckel's diverticulum, internal hernia, Ascariasis, and pregnancy. Other reported conditions include ileal atresia, meconium ileus, enteroenterostomy, leiomyoma of the mesentery, and operations [5]. The suggested mechanism for secondary SBV involves obstruction of a small bowel loop at two fixed points by one of these predisposing conditions. As the loop fills with liquid, peristalsis causes it to twist around its mesentery [6]. In this report, the etiology was the previous pancreas transplant surgery including enteric diversion. The suggested mechanism of the SBV was thought to be the movement of the jejunum to the right lower quadrant from the left upper quadrant during the anastomosis, which caused the rotation of the remaining small intestine segment. Thus, it is likely that the localization of the pancreas and kidney was responsible for this complication. Changing the location by placing the pancreas in the left iliac fossa and the kidney in the right iliac fossa may prevent the rotation of the small intestine in the patients undergoing SPKT. Likewise, bladder drainage to avoid enteric anastomosis is another solution that could prevent this technical complication.

SBV is considered to be a rare condition for patients who are administered exploratory laparotomy for acute abdominal pain. Small bowel volvulus is presented with the classic clinical features of intestinal obstruction including severe abdominal pain as the principal symptom that persists despite routine analgesics, in addition to nausea, vomiting and abdominal distension [7]. Nevertheless, this patient had obscure symptoms which were likely due to the existence of a diabetic condition for a long time. Physical examination may not identify the specific etiology for mild abdominal pain in patients with visceral neuropathy secondary to diabetes mellitus [8]. SPKT transplant cases demand specific clinical expertise that takes into consideration the various difficulties based on the neuropathy of those cases. In cases with a short history, anorexia, vomiting or abdominal distension with a central abdominal pain, we should be increasingly aware of intestinal obstruction, particularly in the postoperative period.

Therapeutic methods are primarily based on causative factors; these options include both simple derotation and resection. However, in the event of a bowel infarction, resection may be necessary. The optimum therapeutic approach for the patients with viable small intestine is uncertain; the alternatives are resection and fixation or simple derotation [5]. If the intestine is gangrenous, almost all authors recommend resection, with or without anastomosis [7]. On the other hand, after the ischemic condition has improved with simple derotation, vascular dilatators (as vasoactive agents) and warm gauze swabs should be kept around the intestines for at least 30 minutes in order to perform the optimal surgical treatment for SBV. Therefore, considering the diabetic state and compromised immunity of our patient, we initially only applied simple derotation with alternative anti-ischemic procedures that included keeping the intestines warm and applying papaverine directly. This procedure may facilitate the resumption of normal bowel circulation. Thus, if reexamination of the intestines reveals significant vascularization in the ischemic bowel segment, the need for the radical surgical intervention can be prevented.

The outcome of SBV is dependent on rapid diagnosis followed by exploratory laparotomy. Because it is so uncommon, it is probable that misdiagnosis accounts for the higher incidence of gangrenous SBV in the Western world [5]. There is a significant difference between the mortality rates of non-gangrenous SBV (5.8% to 8%) [9] and gangrenous SBV (20% to 100%) [10]. In the course of clinical suspicion of intestinal obstruction due to volvulus, intensive preoperative care and low threshold for exploratory laparotomy are recommended to decrease the higher morbidity and mortality rates.

In conclusion, this rare but serious complication resulted in a small bowel obstruction which required prompt surgical intervention. A high level of clinical awareness can ensure low mortality rates. Particularly for general surgeons, small bowel volvulus should always be in differential diagnosis in the patients with a history of previous surgery involving especially intestines. In cases such as this where the patient's clinical symptoms are not clear due to her neuropathy, the length of time between reporting of the symptoms and operation time is important. Although it is still uncertain which surgical treatment is optimal, the etiology, the length of the portion of intestine involved and the blood supply should be considered when choosing the optimum procedure. If rotation involves the majority of the small intestine, the risk of short bowel syndrome is certain, and we should always allow for the possibility that simple derotation will achieve positive results.

## Competing interests

The author(s) declare that they have no competing interests.

## Authors' contributions

**UA **helped perform the operation and was involved in drafting the manuscript and in critical content revision.

**PY **was involved in drafting the manuscript and in critical content revision.

**HT **made substantial contributions to the study's concept and design

**CH **made substantial contributions to the study's concept and gave the final approval of the version to be published

**AC ** was involved in critical content revision and gave the final approval of the version to be published

All authors read and approved the final manuscript
